# Determinants of low socio-economic status and risk of *Plasmodium vivax* malaria infection in Panama (2009–2012): a case–control study

**DOI:** 10.1186/s12936-014-0529-7

**Published:** 2015-01-21

**Authors:** Nicanor Obaldia

**Affiliations:** Department of Immunology and Infectious Diseases, Harvard School of Public Health, Boston, MA USA; Instituto Conmemorativo Gorgas de Estudios de la Salud, Panama, Panama

**Keywords:** *Plasmodium vivax*, Panama, Meso-America, Malaria, Epidemiology, House type, Elimination, Eradication

## Abstract

**Background:**

Identification of risk factors is important for the establishment of malaria elimination programmes tailored to specific regions. Type of house construction had been associated with increasing risk of acquiring malaria. This study aimed at establishing the association between determinants of low socio-economic status (SES) and type of house construction with the likelihood of living in a *Plasmodium vivax* malarious corregimiento (smallest political division) in Panama during 2009–2012.

**Methods:**

To determine the association between type-2 houses (build with deciduous materials) and other determinants of low SES, with living in a malarious corregimiento, this study analyzed demographic and housing census data (2010), and malaria incidence aggregated at the corregimiento level (2009–2012), using a Spearman’s non-parametric correlation test to explore for associations, followed by a case–control study and a reduced multivariate logistic regression approach for confirmation.

**Results:**

A descriptive temporal and spatial analysis indicated that *P. vivax* in Panama was associated with Amerindian reservations. Moreover, this study demonstrated that a strong correlation (deleterious effect) existed between living in a malarious corregimiento and being exposed to a type-2 house (OR = > 1.0) (*p* < 0.001), while, it showed an inverse correlation for exposure to type-1 houses (protective effect) (build with permanent materials) (OR = < 1.0) (*p* < 0.001). In the same way, a significant association between exposure to type-2 houses and the outcome of living in a malarious corregimiento was found using a case–control study approach (Chi^2^ test = *p* < 0.001), that was confirmed applying a reduced multivariate logistic regression fitted model.

**Conclusions:**

This study demonstrated that living in a *P. vivax* malarious corregimiento in Panama during 2009–2012 was strongly correlated with those corregimientos having a high proportion of type-2 houses. A multivariate logistic regression approach at the house and corregimiento level indicated a strong association of type-2 houses, dirt floors and illiteracy with the likelihood of living in a malarious corregimiento. It is expected that these findings will help implement a multi-sectorial approach for the elimination of malaria in poor areas of Panama where malaria is endemic, which emphasizes house improvements such as mosquito-proofing and socio-economic development.

**Electronic supplementary material:**

The online version of this article (doi:10.1186/s12936-014-0529-7) contains supplementary material, which is available to authorized users.

## Background

The recently launched global effort to eradicate malaria has been stimulated by a significant decrease of cases in sub-Saharan Africa [1-8]. With the advent of artemisinin-based combination therapy (ACT), the epidemiological situation of the disease has dramatically changed in some parts of the world; for instance, 10 out of 99 countries where malaria transmission exists have been brought to the brink of elimination [9]. Nonetheless, the burden of malaria remains high among the least developed and poorest areas, and within disadvantaged populations [10]*.* As malaria transmission declines and political commitment and allocation of resources dissipate, identification of risk factors and “hot spots” to target interventions will be increasingly need it.

While *Plasmodium falciparum* is the deadliest of the human malaria parasites; accounting for over 600,000 deaths worldwide each year, mainly in children less than five years old from sub-Saharan Africa [9], *Plasmodium vivax* is a major reason for morbidity within and outside of Africa [8,11]. In the Americas, *P. vivax* malaria remains a major problem in poor underdeveloped areas, predominantly in the Amazonian basin, while in other areas of low transmission, such as Meso-America, it is focalized in rural areas and mostly under control in urban settings [12]. For example, in Panama where malaria transmission have been reduce to pre-elimination levels in the last decade [13], still 90% of malaria cases, mostly all due to *P. vivax* remain focalized to Amerindian reservations — population that only accounts for less than 10% of the total, but where 89.8% of the individuals were living under the poverty line, with less than $ 3.25 US dollars per day by 2012 [14].

Malaria has been correlated with low socio-economic development and poverty [10], and recent evidence suggests, that a causal pathway between poverty and malaria runs in both directions [15]. Furthermore, it has been pointed out that low socio-economic status (SES) doubles the risk of clinical malaria, reducing the capacity of countries to enter the elimination phase and that type of house construction is associated with increasing risk of malaria infection [16-20]. It has also been shown that interventions that address household improvements, such as mosquito-proofing, are associated with a decrease in malaria incidence [16,21-23]. In Latin America, however, only a few studies on the association between type of house building materials and *P. vivax* malaria incidence have been carried out [24].

This study examines the independent association between selected determinants of low SES status including type of house, dirt floors, lack of potable water, lack of electricity, lack of sanitary facilities, unemployment and illiteracy, all determinants of poverty, with the risk of living in a *P. vivax* malarious corregimiento in a low transmission setting; with the hypothesis that in Panama type-2 houses (build with deciduous construction materials), were associated with living in a malarious corregimiento. Results of this study showed a strong statistically significant association between type-2 houses and living in a *P. vivax* malarious corregimiento during 2003–2012. It is expected that this study will help generate new hypothesis and implement public health interventions that addresses risk factors based on a multi-sectorial approach [10], emphasizing house improvements such as mosquito-proofing and socio-economic development of malaria endemic regions.

## Methods

### Study site

This study was carried out in the Republic of Panama. Located in the southern tip of Central America, Panama limits to the east with the Republic of Colombia, the west with Costa Rica, to the north with the Caribbean Sea and to the South with the Pacific Ocean. The country is divided into 10 provinces that comprise 631 corregimientos (smallest political division analog to a county in the USA), with four Amerindian reservations (Ngobe-Bugle, Kuna-Yala, Madugandi-Wargandi and Embera-Wounan); the latter that accounts for only 10% of the ~ 3.4 million inhabitants (2010 Census). Approximately 2/3 of the population of Panama lives in urban or sub-urban areas. The Ministry of Health is divided into 14 health regions that sometimes overlaps a province including: Panama East (EST), Panama West (OES), Panama Metro (MTR) and San Miguelito (MGT); Bocas del Toro (BTO), Chiriqui (CHI), Veraguas (VER), Ngobe-Bugle (NGO), Herrera (HER), Los Santos (LSA), Cocle (CLE), Colon (COL), Darien (DAR) and Kuna-Yala (KUN) (Ministry of Health of Panama, 2010).

### Type of study

This study is based on a ecological case–control study design using publicly available population and housing data (2010 census, Institute of Census and Statistics of the Comptroller office of the Republic of Panama), and a malaria database available from the Panamanian Ministry of Health, Vector Control Programme, comprising 2,295 *P. vivax* cases reported from 631 corregimientos during 2009–2012 in the Republic of Panama. Study variables included in the study were: demographic (total population, gender, age), socio-economic (type of house, dirt floors, lack of potable water, lack of electricity, lack of sanitary facilities, unemployment and illiteracy) and epidemiological (*P. vivax* malaria cases) aggregated at the corregimiento level for the period 2009–2012 (Table 1). For the purpose of this study, type of house was defined as follows: Type-1 = build with permanent construction materials, i.e. cement, wood or clay walls and floors, screened windows, corrugated metal, tile or cemented roofs; Type-2 = build with temporal or deciduous materials, i.e. palm tree roofs and cane walls, raised or at floor level (bohio or rancho); Type-3 = build with cardboard and other materials as temporary shelters as those build by squatters; Type-4 = Individual apartments with private sanitary facilities; Type-5 = Individual apartments or rooms with communal sanitary facilities.

**Table 1:** **Summary of demographic and housing census data (2010 Census, Panama)**

**Population**		**Proportion**
Total	3,405,813	
Male	1,712,584	0.503
Female	1,693,229	0.497
Unemployed	9,427	0.003
Illiterate	7,349	0.002
**Housing**		
Total	896,050	
Type 1*	718,615	0.802
Type 2**	52,935	0.059
Type 3	15,063	0.017
Type 4	83,141	0.093
Type 5	26,296	0.029
Dirt floor	81,268	0.091
w/o/potable water	3,933	0.004
w/o/sanitary	5,444	0.006

Type 1* = Build with permanent materials.

Type 2** = Build with temporal deciduous materials.

w/o = without.

Source: Censo 2010. Instituto Nacional de Estadistica y Censo, Panama.

### Statistical analysis

In order to determine the correlation between independent variables of low SES and type of house, with the risk of *P. vivax* infection by year (incidence) at the corregimiento level, a single Spearman’s non-parametric pair-wise correlation of the study variables was carried out using STATA10® (StataCorp LP, College Station, Texas, USA) or SAS-JMP® (SAS Institute Inc. Cary, NC, USA). Likewise, in order to visualize the trend of the correlations, scattered plots of yearly malaria incidence against proportions of type-1 and type-2 houses by corregimiento were generated using PRISM® (GraphPad Software, Inc., La Jolla, CA, USA) plotting software. Similarly, a case–control study was carried out to determine the association between exposure to type-1-5 households and other determinants of low SES with the outcome of living in a malarious corregimiento. Furthermore, in order to assess the impact of a public health intervention, by removing the exposure of interest on the likelihood of living in a malarious corregimiento, the attributable fraction for the exposed (AttFexp) and the attributable fraction for the population (AttFpop) were calculated for 2009–2012. Finally, to evaluate the association between predictors of low SES and type-2 houses with the likelihood of living in a malarious corregimiento, a multivariate logistic model procedure was applied to estimate the odds of exposure to type-2 houses (binary: yes or no) compared to the odds of not being exposed, on the likelihood of living in a malarious corregimiento (binary: yes or no), controlling for confounders, such as dirt floors (continuous: proportion), lack of potable water (continuous: proportion), lack of sanitary facilities (continuous: proportion), unemployment (continuous: proportion) and illiteracy (continuous: proportion). A malarious corregimiento was defined as a corregimiento that presented one or more cases of *P. vivax* during the study period (2009–2012).

The logistic regression full model was defined as follows: logit (*π*_*i*_) = *ß*_o_ + *ß*_1_Type1 house + *ß*_2_Type-2 house + *ß*_3_*X*_*i*_ + …*ß*_x_*X*_*i*_. Where (*π*_*i*_) is the predicted probability for the ith corregimiento, of being infected with *P. vivax*. The odds of exposure to a type-2 house compared to the odds of not being exposed to a type-2 house, on the likelihood of living in a malarious corregimiento. An odds ratio of 1 mean that the odds for the two groups are the same, indicating that there is no effect. The significance of the OR was assessed using 95% confidence intervals (CI). Using a backward model selection a reduced model including only significant variables at the 0.05 level such as type-2 house, dirt floors and illiteracy was adopted for the final analysis. All statistical analyses were done using STATA10® (StataCorp LP, College Station, Texas, USA). Data were downloaded or transcribed to Filemaker® or Windows® Excel for descriptive and quantitative analysis.

### Spatial distribution

*Plasmodium vivax* malaria incidence and case maps by year (2009–2012) at the corregimiento level were generated using the ArcMap® 10.2 mapping software (Redlands, CA, USA); in a similar way, in order to assess migratory patterns of *P. vivax* cases, 50 selected *P. vivax* cases were used to generate a trace back map using GIS coordinates and connecting lines from the location of the health facility reporting the case to the center of the case self-reported place (corregimiento) of origin using the X to Y toolbar from ArcMap® 10.2.

### Ethics

Data was obtained from publicly available secondary sources and analyzed at the corregimiento level (smallest political division) making impossible to identify the cases, therefore a human subjects protocol was not required.

## Results

### Malaria is focalized to health regions associated with Amerindian reservations

More than 90% of malaria cases including *P. falciparum* and *P. vivax* infections by health region reported between 2003–2011 were found to be associated with the Amerindian reservations of the Ngobe-Bugle (NGO), Kuna-Yala (KUN), Madugandi-Wargandi (EST) and Embera-Wounan (DAR) (Additional files 1 and 2), with female individuals, less than 15 years old, accounting for 60.6% of the *P. vivax* cases compare to 38.3% of males in this age cohort during 2009–2012 (Additional files 3 and 4).

### *Plasmodium vivax* incidence is highest among corregimientos associated with Amerindian reservations

**Figure 1:**
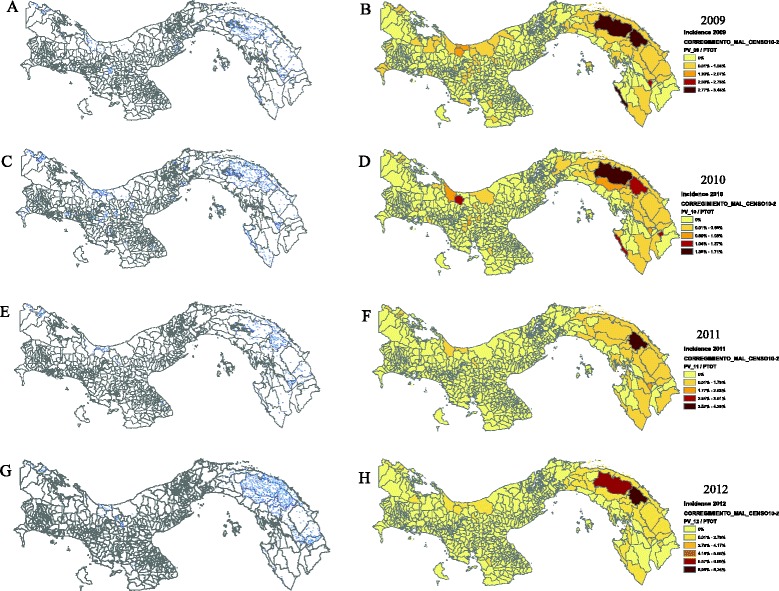
**Individual case and incidence maps by corregimiento of** ***Plasmodium vivax*** **malaria in Panama during 2009–2012. A**, **C**, **E**, **G**) Individual case maps by corregimiento and year. Each dot represents one case. **B**, **D**, **F**, **H**) *P. vivax* malaria incidence maps of Panama by corregimiento and year.

**Figure 2:**
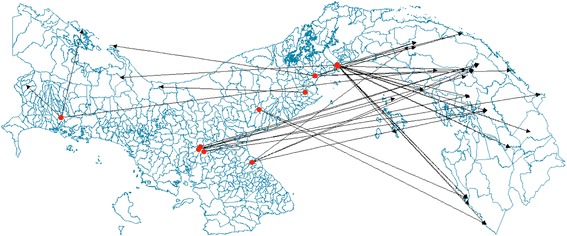
**Corregimiento level map of Panama indicating geo-localization of case catchment and trace-back of fifty** ***Plasmodium vivax*** **cases during 2009–2012 (n = 2295).** Red dots = case catchment regional health center or hospital. Solid line = GPS *x* to *y* coordinates case trace back line. Arrow head = GPS coordinates self reported place of origin.

The incidence of *P. vivax* cases by corregimiento at the country level was estimated to be between < 1 to 8.3% during 2009–2012, with the highest incidence found in those corregimientos associated with the Amerindian reservations of Madugandi-Wargandi in lake Bayano, located in the eastern part of the Province of Panama and west of Darien; Kuna-Yala in the Caribbean coast and the Ngobe-Bugle to the west, including the Provinces of Veraguas and Bocas del Toro with sporadic cases occurring in the provinces of Chiriquí, the Central provinces of Coclé, Herrera and Los Santos, and the provinces of Colon and Panama-west, mainly to east–west migration (Figures 1 and 2); regions with a historical high average rain fall or associated with large bodies of fresh water like lake Bayano (Additional file 5). By 2011 the disease was under control with < 500 cases per year, generally due to *P. vivax* (Additional file 6) and localized to regions with low socio-economic status, usually composed of populations dedicated to subsistence agriculture, or migrant agricultural workers of the coffee, bananas or sugar cane plantations —Populations that migrate every year to the central provinces of Cocle, Herrera and Veraguas during the harvest season; with cases originating as far as the eastern Provinces of Panama and Darien, but also, the western provinces of Bocas del Toro and Chiriquí bordering Costa Rica (Figure 2).

### Determinants of low SES and type-2 houses are associated with increase risk of *P. vivax* infection

**Figure 3:**
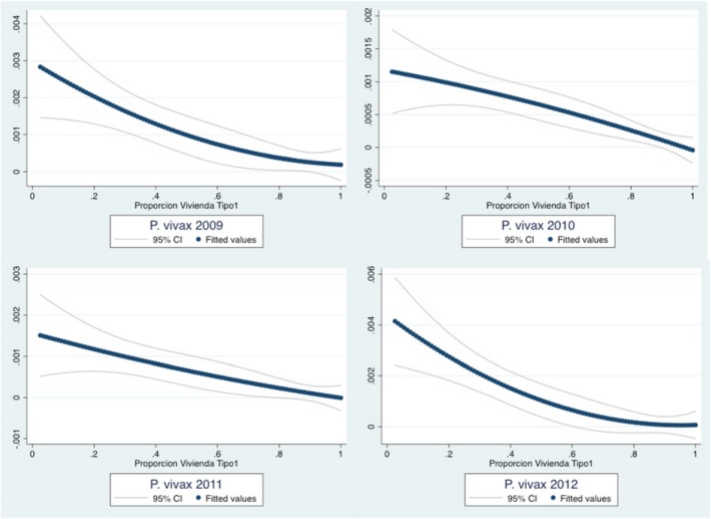
**Scattered plots of yearly malaria infection risk against proportions of type 1 houses by corregimiento in Panama (2009–2012).** Quadratic adjusted scattered and 95% CI plot line; n = 2295 cases, 631 corregimientos.

**Figure 4:**
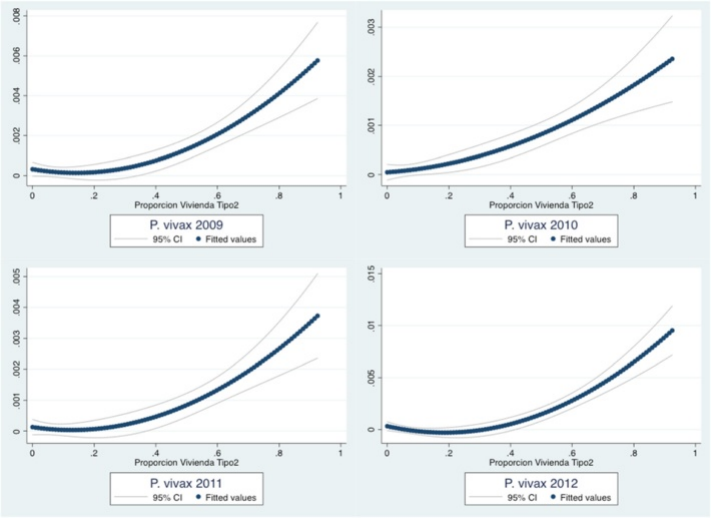
**Scattered plots of yearly malaria infection risk against proportions of type-2 houses by corregimiento in Panama (2009–2012).** Quadratic adjusted scattered and 95% CI plot line; n = 2295 cases, 631 corregimientos.

Results of a single Spearman’s non-parametric pair-wise correlation between determinants of low SES, type of house and malaria incidence by year indicated that those corregimientos with a high proportion of type-1 houses were negatively correlated with malaria incidence (2009–2012), indicating a protective effect of this type of house, while those with a high proportion of type-2 houses were positively correlated, indicating a detrimental effect (*p* < 0.001) (Additional file 7). Scattered plots of yearly malaria infection risk against proportions of type-1 and type-2 houses by corregimiento, showed a negative monotonic quadratic fitted curve between malaria incidence and proportion of type-1 houses and a positive quadratic fitted monotonic curve between incidence and proportion of type-2 houses (Figures 3 and 4).

A case–control study design approach indicated that being exposed to a type-2 house increased the odds of living in a malarious corregimiento 2.14 (2009), 2.43 (2010), 5.04 (2011) and 3.72 (2012) times compare to not being exposed to a type-2 house (Table 2) (Additional file 6). In contrast, for example, exposure to a type-4 house (apartments) reduces the odds of living in a malarious corregimiento in the other direction (1/OR) = 11.1 (2010), 14.28 (2011), 8.33 (2012) and 11.1 (2012) times compare to not being exposed to a type-4 house. The public health impact of removing the exposure to a type-2 house on the likelihood of living in a malarious corregimiento during 2009, 2010, 2011 and 2012 measured by the attributable fraction for the exposed (AttFexp) was 53, 59, 80 and 73% and for the population (AttFpop) 6, 8, 19 and 14% respectively. Nevertheless, the impact of removing the exposure to type-4 houses on the likelihood of not living in a malarious corregimiento was estimated to be for the AttFexp 91, 93, 88 and 91% and for the AttFpop 10, 10, 10 and 9% during 2009, 2010, 2011 and 2012, respectively (Table 2).

**Table 2:** **Case–control study odds and 95% confidence intervals on the likelihood of living in a malarious corregimiento given exposure to determinants of low SES and type of house in Panama during 2009-2012**

**Odds ratio (95% confidence interval)**																
	**2009**				**2010**				**2011**				**2012**			
	**OR**	**CI 95%**	**AttFexp**	**AttFpop**	**OR**	**CI 95%**	**AttFexp**	**AttFpop**	**OR**	**CI 95%**	**AttFexp**	**AttFpop**	**OR**	**CI 95%**	**AttFexp**	**AttFpop**
**Total houses**	1.00				1				1				1			
Type 1	**1.40**	(1.38,1.42)	0.29	0.24	**1.21**	(1.19,1.23)	0.17	0.14	**0.61**	(0.60,0.63)	0.39	0.31	**0.84**	(0.82,0.85)	0.16	0.13
Type 2	**2.43**	(2.39,2.48)	0.59	0.06	**2.74**	(2.68,2.79)	0.63	0.08	**5.52**	(5.40,5.64)	0.82	0.19	**4.15**	(4.06,4.24)	0.76	0.14
Type 3	**1.07**	(1.03,1.12)	0.07	0.00	**1.15**	(1.10,1.21)	0.13	0.00	**1.24**	(1.16,1.33)	0.19	0.00	**1.41**	(1.34,1.49)	0.29	0.01
Type 4	**0.09**	(0.09,0.98)	0.91	0.10	**0.07**	(0.07,0.08)*	0.93	0.10	**0.12**	(0.11,0.13)	0.88	0.09	**0.09**	(0.09,0.1)	0.91	0.09
Type 5	**0.55**	(0.53,0.57)	0.45	0.01	**0.59**	(0.56,0.61)	0.41	0.13	**0.69**	(0.64,0.73)	0.31	0.01	**0.42**	(0.39,0.45)	0.58	0.02
Dirt Floor	**1.47**	(1.44,1.49)	0.32	0.04	**1.61**	(1.58,1.64)	0.38	0.05	**2.68**	(2.61,2.74)	0.63	0.13	**2.32**	(2.27,2.37)	0.57	0.10
w/o potable water	**1.97**	(1.94,2.01)	0.49	0.06	**1.57**	(1.53,1.60)	0.36	0.04	**3.14**	(3.06,3.21)	0.68	0.13	**2.44**	(2.39,2.50)	0.59	0.09
w/o sanitary	**1.63**	(1.61,1.66)	0.39	0.07	**1.87**	(1.84,1.90)	0.95	0.17	**2.94**	(2.88,3.00)	0.66	0.19	**2.34**	(2.30,2.39)	0.57	0.10
**Total population**	1.00				1.00				1.00				1.00			
Unemployed	**0.95**	(0.94,0.97)	0.05	0.00	**0.86**	(0.84,0.87)	0.14	0.00	**0.76**	(0.74,0.78)	0.24	0.01	**0.80**	(0.78,0.82)	0.20	0.01
Illiterate	**1.77**	(1.75,1.79)	0.44	0.03	**1.80**	(1.78,1.83)	0.45	0.03	**2.81**	(2.77,2.85)	0.64	0.07	**2.43**	(2.40,2.47)	0.59	0.05
Female	1.00				1.00				1.00				1.00			
Male	**1.05**	(1.04,1.05)	0.04	0.02	**1.03**	(1.03,1.04)	0.03	0.02	**1.04**	(1.04,1.06)	0.05	0.02	**1.09**	(1.08,1.10)	0.08	0.04

**Bold face** = Chi test p < 0.001; * = p = 0.001.

AttFexp = Atributable fraction expose.

AttFPop = Atributable fraction population.

w/o = without.

Results of a full multivariate logistic regression model to determine the odds ratio (OR) of exposure to determinants of low SES and type of house compared to not being exposed, on the likelihood of living in a malarious corregimiento is shown in Table 3. In general, results of the full model indicated that the odds of exposure to type-2 houses, dirt floors and illiteracy were statistically significant at the *p* < 0.05 level, while, type-1 houses, without potable water, without electricity, unemployment and illiteracy were not (*p* > 0.05), all other confounders held constant. Results of the reduced model including only type-2 house, dirt floors and illiteracy covariates as shown in Table 3, revealed that the odds of living in a malarious corregimiento, if expose to a type-2 house, were about 11.19 (2009), 33.77 (2010), 99.19 (2011) and 67.2 (2012) (*p* < 0.001) times the corresponding odds to not being exposed to a type-2 house, all other confounders held constant. In a similar way, exposure to illiteracy, dramatically increased the odds on the likelihood of living in a malarious corregimiento compared to not being exposed to illiteracy, though the confidence intervals for this exposure were extremely broad, making this estimation unreliable (Table 3). In contrast, the odds of exposure to dirt floors were strongly protective (Table 3). Interaction terms (Type-2 house x dirt floors or type-2 house x illiteracy) did not show a statistical significant effect modification of the study variables and were not included in the table. Except for illiteracy during 2010 that was borderline significant (*p* = 0.073), all the other variables were statistically significant every year.

**Table 3:** **Multivariate logistic regression model odds of exposure to selected determinants of socio-economic status and type of house on the likelihood of living in a malarious corregimiento in Panama during 2009-2012**

**Full model**								
	**2009**		**2010**		**2011**		**2012**	
**Variable**	**OR**	***P***	**OR**	***P***	**OR**	***P***	**OR**	***P***
Type-1 house	3.24	0.478	10.42	0.356	8.05	0.566	5.65	0.575
Type-2 house	**43.63**	**0.038**	**847.63**	**0.012**	**1388.21**	**0.050**	**735.60**	**0.038**
Dirt floor	**0.12**	**0.002**	**0.09**	**0.006**	**0.08**	**0.019**	**0.09**	**0.013**
Without potable water	0.54	0.377	0.30	0.158	3.42	0.175	1.42	0.692
Without sanitary	1.47	0.586	0.61	0.594	0.08	0.072	0.14	0.088
Without electricity	0.93	0.869	0.80	0.696	0.68	0.672	0.90	0.871
Unemployed	0.04	0.780	8.0X10^-3^	0.612	1.52x10^-15^	0.106	2.0x10^-9^	0.271
Illiterate	**1283.64**	**0.009**	**2257.14**	**0.018**	**5398.53**	**0.034**	**1.64x10** ^**4**^	**0.010**
**Reduced model**								
	**2009**		**2010**		**2011**		**2012**	
	**OR**	***P***	**OR**	***P***	**OR**	***P***	**OR**	***P***
Type-2 house	**11.19**	**0.001**	**33.77**	**0.000**	**99.19**	**0.000**	**67.2**	**0.000**
Dirt floor	**0.14**	**0.001**	**0.08**	**0.001**	**0.04**	**0.000**	**0.53**	**0.000**
Illiterate	**1065.83**	**0.004**	139.04	0.073	**1186.47**	**0.032**	**1989.9**	**0.015**

**Bold face** = *p* < 0.05.

## Discussion

This study demonstrates the association between selected determinants of low socio-economic status such as type-2 houses (build with temporary or deciduous construction materials), dirt floors and illiteracy with the likelihood of living in a *P. vivax* malarious corregimiento (smallest political division) during 2009–2012 in Panama.

In the same way, the study suggests that Panama is in a pre-elimination stage and that malaria is focalized to health regions (“hot spots”) associated with Amerindian reservations; where ~ 90% of malaria cases including both *P. falciparum* and *P. vivax* infections occurred during 2003–2012. This observation is supported by a focal low incidence of malaria by corregimiento (<1 – 8.3%) observed during 2009–2012. For instance, by 2011 the disease was under control with less than 500 cases per year, mostly due to *P. vivax* and localized to regions associated with Amerindian reservations (Figure 1).

However, as economic development reaches these focal areas of active malaria transmission, people tend to migrate looking for better jobs or education, bringing along parasites that seed new transmission foci to areas where malaria had been previously eliminated. Interestingly, part of the population of these reservations are dedicated to subsistence agriculture, but others are seasonal migrant agricultural workers dedicated to labour in the coffee, bananas or sugar cane plantations —Populations that migrate westward to the highlands of the province of Chiriquí for the coffee harvest season, or to the central provinces of Coclé, Veraguas and Herrera to harvest sugar cane. Using GIS mapping software 50 *P. vivax* cases were trace back to their places of origin, detecting movement of cases from as far as the eastern Provinces of Panama and Darien, but also, the western provinces of Bocas del Toro and Chiriquí bordering Costa Rica, towards the central provinces and to Panama City where malaria has been eliminated. These highly movable populations will have to be further investigated to account for other patterns of internal, continental and extra-continental illegal immigration at the moment of designing malaria control and eradication strategies (Figure 2).

Intriguingly, when *P. vivax* cases were stratified by age and gender for the period 2009–2012, the study found that female individuals less than 15 years old presented the highest number of *P. vivax* cases (60.6%) compared to males (38.3%). This finding contrast with recent reports from a moderate transmission setting in Tanzania, where male individuals in this same age cohort presented the highest malaria seroprevalence [25]. However, anecdotal reports indicate that girls in the Kuna reservation of Madugandi-Wargandi in lake Bayano, engage in leisure and domestic activities such as sewing, laundry and cooking near or around the household, while young males in this age cohort are dedicated mainly to agricultural, hunting or fishing activities away from home. Activities that are in part associated with the lake and rivers banks, where houses are prevalently located —both areas of known breeding sites for *Anopheles albimanus* mosquitos [26]; exposing them differentially to the vector [27]. This finding suggests differential malaria transmission based on age, gender and cultural or behavioural risk factors that should be further investigated.

In areas where malaria is endemic, house design and building are based on local experience, customs and available materials [28]. Type-2 houses —the most prevalent type of house in the Amerindian reservations of Panama—, known locally as “bohíos” or “ranchos”, are build based on the palm tree and local experience, usually over dirt floors or raised above ground in stilts, to avoid seasonal flooding or insect nuisance [29]. This type of house don’t offer much protection against the elements or vectors of disease unless are raised above ground [29], or its occupants use a mosquito net, but allows air flow for comfort. For example, a recent survey at the household level in The Gambia showed that children from the poorest quintiles living in houses build with poor walls, roofs and windows construction materials, were associated with a high prevalence of malaria [15], though, in the general population only poor wall housing materials were associated with a high prevalence.

The fact that during a recent survey at the Madugandi reservation in lake Bayano, only 7% of households reported using mosquito nets [Caceres L, Rovira J, Torres R, Calzada JE, Victoria C, Griffith M: Conocimientos, actitudes y practicas sobre la malaria en la poblacion indigena guna de la Comarca de Madugandi, Panama. Submitted, 2014], might help explain why in this study type-2 houses being the most prevalent in the Amerindian reservations of Panama, were highly correlated and associated to *P. vivax* malarious corregimientos. Suggesting that transmission occurs inside or closely associated with the house. Albeit, *A. albimanus* is predominantly exophagic and exophilic, there is evidence in the region of indoor resting after feeding [27]. In the same way, the strong association between illiteracy and *P. vivax* infection might be explain by a 30% illiteracy [Caceres L, Rovira J, Torres R, Calzada JE, Victoria C, Griffith M: Conocimientos, actitudes y practicas sobre la malaria en la poblacion indigena guna de la Comarca de Madugandi, Panama. Submitted, 2014] and 69.5% extreme poverty rates reported for the Amerindian reservations in recent surveys [14]. In contrast, the indication that dirt floors were protective of living in a *P. vivax* malarious corregimiento remains unclear. For instance, only 6% of the houses at the Madugandi reservation have cement floors [Caceres L, Rovira J, Torres R, Calzada JE, Victoria C, Griffith M: Conocimientos, actitudes y practicas sobre la malaria en la poblacion indigena guna de la Comarca de Madugandi, Panama. Submitted, 2014], Though, other confounding factors cannot be ruled out [30].

A limitation of this study is that it uses aggregated data at the corregimiento level for the multivariate correlations and logistic regression models and even though several confounding variables were adjusted for and the universe was the total houses and population of Panama, there could still be residual confounding from variables not accounted for. Moreover, ecologic study designs are prompt to the “ecological fallacy” when aggregated data (Group level) findings are generalized to the individual level [31,32]. The results of this study should be interpret interpreted with caution, though are useful preliminary indicators to causal hypothesis despite its methodological limitations when presented as a population level study because [33,34]: First, they are inexpensive and take little time to implement due to the availability of secondary sources (i.e. census databases); second, they are fast to analyse when compare to more elaborate and expensive individual level study designs (i.e. surveys). Moreover, they are particularly relevant when evaluating social processes or interventions at the population level (new programs, policy or legislation) [33]. However, if the outcome at the group level is classified as dichotomous (as in the case–control study presented here) and the inferences are at the group level the study is not an ecologic study [34].

## Conclusions

In the Americas, malaria remains a major problem in regions of low SES. In order to achieve elimination, current strategies must have a multi-sectorial approach that addresses social and environmental determinants of malaria infection. The current focal low transmission setting of malaria in Panama presents a unique opportunity to study the association between determinants of low SES and malaria at the population level to help guide interventions, policy and legislation. This study suggests that Panama is in a malaria pre-elimination stage and that transmission is low, focalized and mainly restricted to the Amerindian reservations. Furthermore, that type of house building materials and design play an important role in the transmission of *P. vivax* malaria in these endemic “hot-spots”. Increase risk of malaria infection was associated with those corregimientos having a high exposure to type-2 houses. Exposure to type-2 houses strongly increases the likelihood of living in a malarious corregimiento compare to not being exposed to a type-2 house. Type-2 houses, dirt floors and illiteracy were associated with the likelihood of living in a malarious corregimiento. Removing these exposures will likely contribute to accelerate malaria elimination in Panama. These results suggest that house improvements such as mosquito proofing and economic development of these areas may play an important role in a multi-sectorial approach for the control and elimination of malaria in Panama.

## Additional files

Additional file 1:
**Number of malaria cases by health region in Panama during 2003–2011.** A) *Plasmodium falciparum.* B) *Plasmodium vivax*. Additional file 2:
***Plasmodium vivax*** **infections by health region in Panama during 2003–2010.** Bocas del Toro = BTO; Chiriqui = CHI; Cocle = CLE; Colon = COL; Darien = DAR; Herrera = HER; Kuna-Yala = KUN; Los Santos = LSA; Ngobe-Bugle = NGO; Panama East = EST, Panama West = OES; Panama Metro = MTR; San Miguelito = MGT and Veraguas = VER. Additional file 3:
**Number of** ***Plasmodium vivax*** **cases by gender and age group in Panama during 2009–2012.** n = 2295 cases. Additional file 4:
**Summary of malaria cases by gender and age group in Panama 2009–2012.**
Additional file 5:
**Historic average rainfall and** ***Plasmodium vivax*** **incidence maps of Panama: A) Historic average rainfall mm (1971–2002).** B) Overlay map of individual *P. vivax* malaria cases in Panama (2009–2012) at the corregimiento level and historic average rainfall mm (1971–2002). Each dot represents one case. (Historic average rainfall map adapted from the Ministry of Health, Panama). Additional file 6:
**Case–control summary data of malaria exposure by type of house and selected determinants of low SES in Panama during 2009–2012.**
Additional file 7:
**Spearman's nonparametric correlation matrix of malaria incidence by corregimiento against determinants of low socio-economic status and type of house in Panama during 2009-2012.**

## References

[1] Bassat Q, Alonso PL (2011). Defying malaria: fathoming severe *Plasmodium vivax* disease. Nat Med..

[2] The malERA CGoM. A research agenda for malaria eradication: modeling. PLoS Med. 2011;8:1–9. 10.1371/journal.pmed.1000403 PMC3026697 21283605

[3] Editorial. Is malaria eradication possible? Lancet. 2007;370:1459. 10.1016/S0140-6736(07)61609-2 17964329

[4] Alonso PL, Brown G, Arevalo-Herrera M, Binka F, Chitnis C, Collins F (2011). A research agenda to underpin malaria eradication. PLoS Med..

[5] Greenwood BM, Fidock DA, Kyle DE, Kappe SH, Alonso PL, Collins FH (2008). Malaria: progress, perils, and prospects for eradication. J Clin Invest..

[6] Mendis K, Rietveld A, Warsame M, Bosman A, Greenwood B, Wernsdorfer WH (2009). From malaria control to eradication: the WHO perspective. Trop Med Int Health..

[7] Roberts L, Enserink M (2007). Malaria. Did they really say … eradication?. Science..

[8] Breman JG, Brandling-Bennett AD (2011). The challenge of malaria eradication in the twenty-first century: research linked to operations is the key. Vaccine..

[9] WHO. World Malaria Report. Geneva: World Health Organization; 2012.

[10] UNDP RBMP (2013). Multisectorial Action Framework for Malaria.

[11] Mendis K, Sina BJ, Marchesini P, Carter R (2001). The neglected burden of *Plasmodium vivax* malaria. Am J Trop Med Hyg..

[12] Arevalo-Herrera M, Quinones ML, Guerra C, Cespedes N, Giron S, Ahumada M (2012). Malaria in selected non-Amazonian countries of Latin America. Acta Trop..

[13] Obaldia N, 3rd, Baro NK, Calzada JE, Santamaria AM, Daniels R, Wong W, Chang HH, Hamilton EJ, Arevalo-Herrera M, Herrera S, Wirth DF, Hartl DL, Marti M, Volkman SK: Clonal outbreak of *Plasmodium falciparum* infection in Eastern Panama. J Infect Dis. 2014. First published online October 21, 2014 doi:10.1093/infdis/jiu575 10.1093/infdis/jiu575 PMC4366603 25336725

[14] Dieguez J, Alvarado R: Indigencia y pobreza: encuesta de mercado de trabajo. Panama, Republica de Panama, Ministerio de Economia y Finanzas ed. pp. 17. 2012.

[15] Sonko ST, Jaiteh M, Jafali J, Jarju LB, D’Alessandro U, Camara A (2014). Does socio-economic status explain the differentials in malaria parasite prevalence? Evidence from The Gambia. Malar J..

[16] Lindsay S, Jawara M, Paine K, Pinder M, Walraven G, Emerson PM (2003). Changes in hosue design reduce exposure to malaria. Trop Med Int Health..

[17] Gamage-Mendis A, Carter R, Mendis C, De Zoysa A, Herath P, Mendis K (1991). Clustering of malaria infections within an endemic populations: risk of malaria associated with type of housing construction. Am J Trop Med Hyg..

[18] Yamamoto S, Louis VR, Sie A, Sauerborn R (2010). Household risk factors for clinical malaria in a semi-urban area of Burkina Faso: a case–control study. Trans R Soc Trop Med Hyg..

[19] Liu JX, Bousema T, Zelman B, Gesase S, Hashim R, Maxwell C (2014). Is housing quality associated with malaria incidence among young children and mosquito vector numbers? Evidence from Korogwe, Tanzania. PLoS One..

[20] Konradsen F, Amerasinghe P, van der Hoek W, Amerasinghe F, Perera D, Piyaratne M (2003). Strong association between house characteristics and malaria vectors in Sri Lanka. Am J Trop Med Hyg..

[21] Lindsay S, Emerson PM, Charlwood J (2002). Reducing malaria by mosquito-proofing houses. Trends Parasitol..

[22] Ye Y, Hoshen M, Louis V, Seraphin S, Traore I, Sauerborn R (2006). Housing conditions and *Plasmodium falciparum* infection: protective effect of iron-sheet roofed houses. Malar J..

[23] Bradley J, Rehman AM, Schwabe C, Vargas D, Monti F, Ela C (2013). Reduced prevalence of malaria infection in children living in houses with window screening or closed eaves on Bioko Island, equatorial Guinea. PLoS One..

[24] Danis-Lozano R, Rodriguez MH, Betanzos-Reyes AF, Hernandez-Avila JE, Gonzalez-Ceron L, Mendez-Galvan JF (2007). Individual risk factors for *Plasmodium vivax* infection in the residual malaria transmission focus of Oaxaca, Mexico. Salud Publica Mex..

[25] Mosha JF, Sturrock HJ, Brown JM, Hashim R, Kibiki G, Chandramohan D (2014). The independent effect of living in malaria hotspots on future malaria infection: an observational study from Misungwi. Tanzania Malar J..

[26] Caceres L, Rovira J, Garcia A, Torres R (2011). Determinacion de la resistencia a insecticidas organofosforados, carbamatos y piretroides en *Anopheles albimanus* (Diptera: Culicidae) de Panama. Biomedica..

[27] Mullen G, Durden L (2009). Medical and Veterinary Entomology.

[28] Knudsen J, von Seidlein L (2014). Healthy Homes in Tropical Zones: Improving Rural Housing in Asia and Africa.

[29] Charlwood JD, Pinto J, Ferrara PR, Sousa CA, Ferreira C, Gil V (2003). Raised houses reduce mosquito bites. Malar J..

[30] Kleinbaum D, Kupper L, Nizam A, Muller K (2008). Applied regression analysis and other multivariable methods.

[31] Schwartz S (1994). The fallacy of the ecological fallacy: the potential misuse of a concept and the consequences. Am J Public Health..

[32] Diez-Roux AV (1998). Bringing context back into epidemiology: variables and fallacies in multilevel analysis. Am J Public Health..

[33] Morgenstern H (2008). Ecologic Studies.

[34] Dohoo I, Martin W, Stryhn H (2009). Ecological and Group Level Studies.

