# Supplementary figures and images for: Determinants of low socio-economic status and risk of Plasmodium vivax malaria infection in Panama (2009–2012): a case–control study
Source: Malar J. 2015 Jan 21;14:14. doi: 10.1186/s12936-014-0529-7 (PMC4320569; doi:10.1186/s12936-014-0529-7)

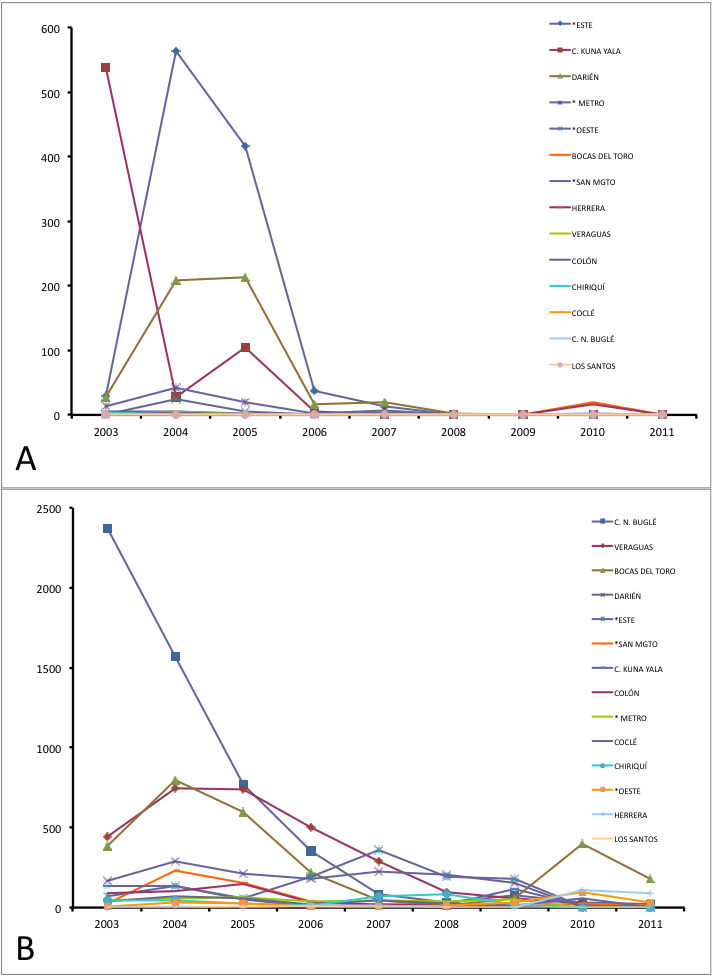

Supplement: Additional file 1: — Number of malaria cases by health region in Panama during 2003–2011. A) Plasmodium falciparum. B) Plasmodium vivax. [file 12936_2014_529_MOESM1_ESM.png]

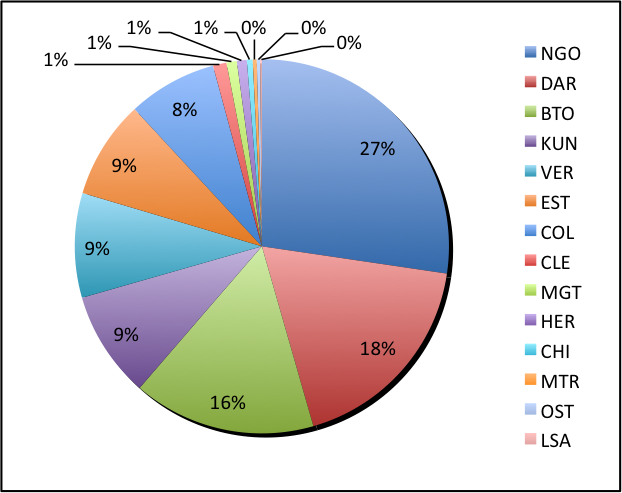

Supplement: Additional file 2: — Plasmodium vivax infections by health region in Panama during 2003–2010. Bocas del Toro = BTO; Chiriqui = CHI; Cocle = CLE; Colon = COL; Darien = DAR; Herrera = HER; Kuna-Yala = KUN; Los Santos = LSA; Ngobe-Bugle = NGO; Panama East = EST, Panama West = OES; Panama Metro = MTR; San Miguelito = MGT and Veraguas = VER. [file 12936_2014_529_MOESM2_ESM.png]

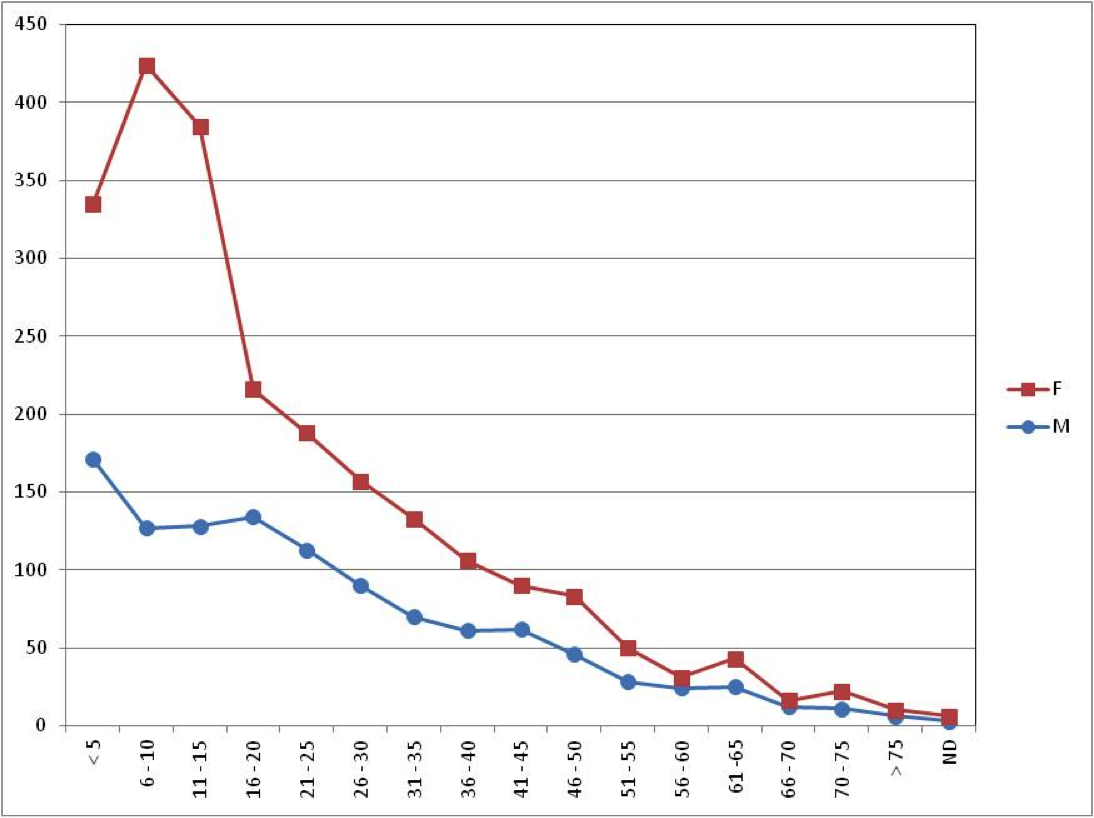

Supplement: Additional file 3: — Number of Plasmodium vivax cases by gender and age group in Panama during 2009–2012. n = 2295 cases. [file 12936_2014_529_MOESM3_ESM.png]

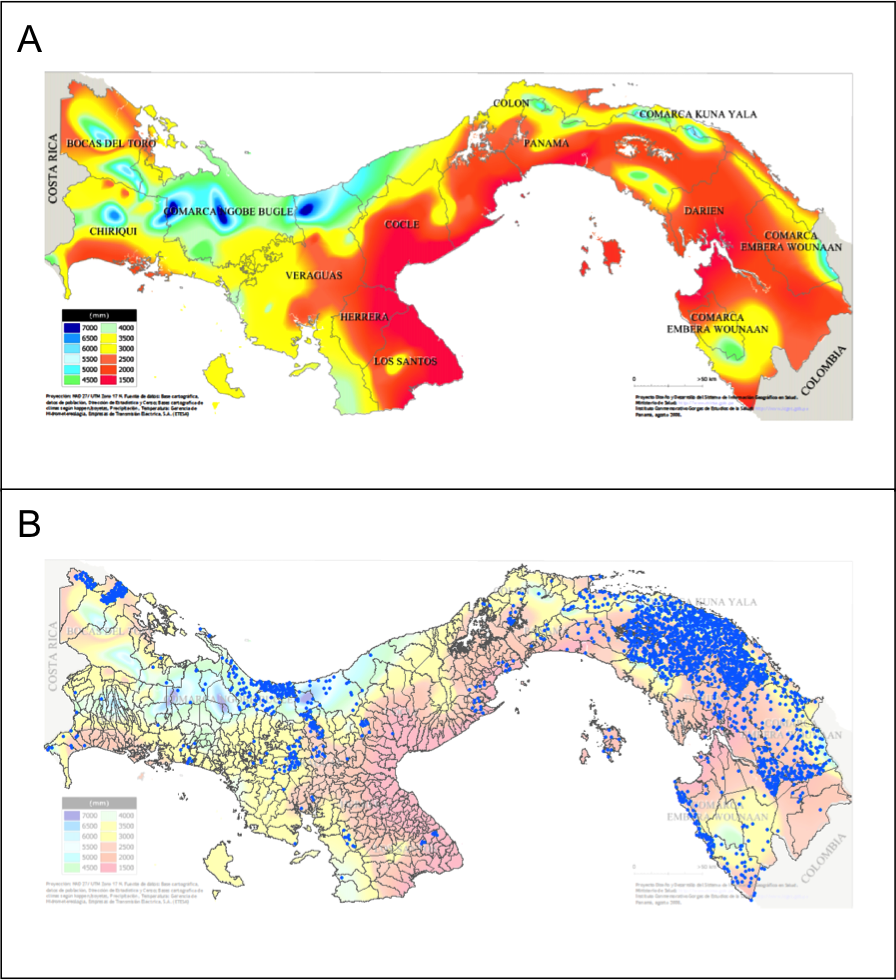

Supplement: Additional file 5: — Historic average rainfall and Plasmodium vivax incidence maps of Panama: A) Historic average rainfall mm (1971–2002). B) Overlay map of individual P. vivax malaria cases in Panama (2009–2012) at the corregimiento level and historic average rainfall mm (1971–2002). Each dot represents one case. (Historic average rainfall map adapted from the Ministry of Health, Panama). [file 12936_2014_529_MOESM5_ESM.png]
